# Three-dimensional curvature mismatch of the acetabular radius to the femoral head radius is increased in borderline dysplastic hips

**DOI:** 10.1371/journal.pone.0231001

**Published:** 2020-04-06

**Authors:** Tohru Irie, Alejandro A. Espinoza Orías, Tomoyo Y. Irie, Shane J. Nho, Daisuke Takahashi, Norimasa Iwasaki, Nozomu Inoue

**Affiliations:** 1 Department of Orthopedic Surgery, Rush University Medical Center, Chicago, IL, United States of America; 2 Department of Orthopaedic Surgery, Faculty of Medicine and Graduate School of Medicine, Hokkaido University, Sapporo, Japan; Assiut University Faculty of Medicine, EGYPT

## Abstract

Whether borderline hip dysplasia is pathologic remains unclear. In order to evaluate the three-dimensional joint congruity, this study sought to answer the question: are borderline dysplastic hip curvature mismatch and eccentricity between the acetabulum and the femoral head different from dysplastic or control hips three-dimensionally? The 113 hips, categorized as: dysplastic (LCEA ≤ 20°), 47 hips; borderline (20° ≤ LCEA < 25°), 32 hips; and control (25° ≤ LCEA < 35°), 34 hips; were evaluated. Three-dimensional (3D) femoral and coxal bone models were reconstructed from CT images. Using a custom-written Visual C++ routine, the femoral head and acetabular radii of curvature, and the femoral head and the acetabular curvature center were calculated. Then the ratio of the acetabular radius to the femoral head radius (3D curvature mismatch ratio), and the distance between the acetabular curvature center and the femoral head center (3D center discrepancy distance) were calculated. These indices were compared statistically among the three groups using Tukey’s *post hoc* test. The mean 3D curvature mismatch ratio in the borderline (1.13 ± 0.05) was smaller than in the dysplasia (1.23 ± 0.08, p < 0.001), and larger than in the control (1.07 ± 0.02, p < 0.001). The mean 3D center discrepancy distance in the borderline (3.2 ± 1.4 mm) was smaller than in the dysplasia (4.8 ± 2.3, p < 0.001) and larger than in the control (1.6 ± 0.7, p < 0.001). These results demonstrated that three-dimensional congruity of the borderline dysplastic hip is impaired, but its incongruity is not as severe as in dysplastic hips. The 3D curvature mismatch ratio and the 3D center discrepancy distance can be valuable signs of joint congruity in patients with borderline dysplasia. However, future studies are necessary to clarify any associations between curvature mismatch and pathogenesis of osteoarthritis in borderline dysplasia.

## Introduction

Approximately 90% of the cases of adult hip osteoarthritis (OA) are associated with some developmental abnormalities [1,2]. Considering the natural history of moderate to severe developmental dysplasia of the hip (DDH), it has been well accepted that there is an association between DDH and OA [3,4]. Although the pathophysiology of DDH is not fully understood, hips with a lateral center-edge angle (LCEA) of less than 20° were defined as dysplastic hip by Wiberg [5] and are recognized as pathologic. In general, hips with an LCEA of larger than 25° are recognized as non-dysplastic hip [5]. On the other hand, there is a transition zone so-called “borderline dysplasia” between dysplastic and non-dysplastic hips. Since little attention has been given to borderline dysplasia until the introduction of hip arthroscopic procedures, the natural history of borderline dysplasia is not fully understood. Therefore, we do not know its association to OA pathogenesis as well as in the case of frank dysplasia, which is far from being completely understood, even with it being an older known clinical entity.

As a fundamental question, whether borderline dysplasia is pathologic or not, remains unclear. In patients with DDH, loss of joint congruity has been recognized as an important initiator of OA [6–8]. Loss of congruity can cause damage of the acetabular labrum and cartilage and ultimately lead to OA [8–10]. Furthermore, labrum tears and cartilage damages are often observed in borderline dysplastic hips as well, adding more confusion to the picture [11–14].

The hip joint is considered to be a highly constrained ball and socket joint, which means that a hemispherical acetabulum surface and its corresponding spherical femoral head possess tight three-dimensional joint congruity. The concept and definition of excellent congruency proposed by Yasunaga et al., which has been widely accepted [15], is to use the curvature of the acetabulum and femoral head seen in anteroposterior (AP) pelvic radiographs because they are almost identical. Meanwhile, the commonly used two-dimensional measures of hip congruency have low intra-observer and inter-observer reliability [16,17]. When evaluating the spectrum of hip dysplasia, three-dimensional evaluation can provide much more useful information than two-dimensional evaluation alone [18,19]. Since the congruity condition affects the hip natural history or treatment strategy, evaluation of congruity from a three-dimensional point of view is clinically critical.

When both acetabulum and femoral head constitute a congruent joint, the arc of the femoral head matches the arc of the acetabulum with a consistent joint space throughout [16,17]. In theory, the mismatch between the three-dimensional acetabular radius and the femoral head radius can reflect loss of three-dimensional joint congruity. In such a case, the positional relationship between the acetabular curvature center and the femoral head center can be eccentric. Actually, the center gap (distance between the acetabular rotation center and the femoral head center) has been reported as an independent risk factor for OA progression [3]. Nevertheless, quantitative three-dimensional congruity evaluation is still a challenge, mostly due to the lack of the availability of proper methods for this purpose.

Structural configurations in normal, borderline dysplastic, and frank dysplastic hips exist within a broad continuum. In order to elucidate the pathophysiology of borderline dysplasia, it is essential to evaluate the three-dimensional joint congruity and compare that against the extremes of said continuum: the frank dysplastic hip and the “normal” (or more properly described as *non-pathological*) hip.

With the evaluation of three-dimensional joint congruity in mind, this study sought to answer the following two questions: (1) How much correlation exists between the two-dimensional (femoral head and acetabular radii measurements based on AP pelvic radiographs) and the three-dimensional measurements based on CT?, (2) Do three-dimensional curvature mismatch and joint eccentricity between the acetabulum and the femoral head show differences when comparing dysplastic, borderline dysplastic and control hips?”

## Materials and methods

### Patients and study design

The study was approved by the ethics committee of the Hokkaido University Hospital (approval number 019–0131), and written informed consent was provided by participants for their clinical records to be used. We evaluated 165 contralateral hips of patients aged 16 to 60 who underwent one of three types of surgeries between January 2013 and April 2018 at our institution: a) eccentric rotational acetabular osteotomy (ERAO) for DDH, b) curved intertrochanteric varus osteotomy (CVO) for idiopathic osteonecrosis of the femoral head (ONFH), and c) total hip replacement (THR) for ONFH or OA.

All patients underwent bilateral hips CT scans, MRIs, and supine AP pelvic radiographs taken by Siebenrock’s standardized technique [20]. CT images, MRIs, and AP pelvic radiographs were obtained within 7 days for each patient. LCEA of Wiberg [5], acetabular roof obliquity (ARO) [21], radiographic OA were evaluated by AP radiographs. A break in the Shenton line on AP radiographs larger than 5 mm was defined as joint subluxation [9]. Acetabular anteversion angle was determined in the axial plane passing through the femoral head center as the angle formed by the intersection of a line connecting the anterior and posterior edges of the acetabulum and a sagittal line [19]. ONFH was confirmed by both AP radiographs and MRIs. Cartilage thinning was evaluated by MRI and defined as radiographic OA (Kellgren-Lawrence Grade 1).

Exclusion criteria were: (1) LCEA ≥ 35°; (2) ipsilateral prior hip surgery or trauma; (3) subluxation; (4) aspherical femoral head; (5) ipsilateral ONFH; or (6) radiographic OA (Kellgren-Lawrence Grade 1, 2, 3 or 4). The remaining hips were categorized based on LCEA as follows: dysplasia group, LCEA < 20°; borderline group, 20° ≤ LCEA < 25°; and control group.

### Evaluation of two-dimensional femoral head and acetabular radii of curvature

The femoral head center was identified with a spherical template on digital AP pelvic radiographs by placing the template congruent with the aspect of the femoral head contained by the acetabulum [22] using an image analysis system (ZioCube, Ziosoft, Tokyo, Japan). The two-dimensional (2D) femoral head radius of curvature was calculated based on the radius of the fitted template. Similarly, the acetabular center was identified by fitting a spherical template to the acetabular sourcil on digital AP pelvic radiographs. The 2D acetabular radius of curvature was calculated based on the radius of the fitted template.

### Three-dimensional model creation and calculation of three-dimensional curvature mismatch ratio and center discrepancy

The analysis was based on the retrospective evaluations of CT scans underwent for preoperative examinations. All patients underwent CT scans (CT High Speed Advantage; GE Medical Systems, Milwaukee, WI, USA) in the supine position with an imaging interval ranging from the entire pelvis to the lower level of tibial tuberosity. Positioning of patients in the scanner was standardized with their hips and knees fully extended, the legs in neutral abduction/adduction and with the patellae pointing directly upwards. Using the obtained CT images, the realistic hip neutral extension/flexion and neutral abduction/adduction were confirmed based on the alignment of the entire femur and the entire pelvis. The hip neutral internal/external rotation was also confirmed from the position of the pelvis and the neutral rotation of the knee joints. Slice thickness and interval were set at 1 mm each. CT images of each hip joint were imported in DICOM format and segmented using commercially-available segmentation software (Mimics ver. 21, Materialise, Leuven, Belgium), resulting in three-dimensional femoral and coxal bone models which were later exported as point-cloud and polygon mesh models using the same software application. These models were then processed with a custom-written program created in Microsoft Visual C++ with Microsoft Foundation Class programming environment (Microsoft, Redmond, WA) [23,24].

**Fig 1:**
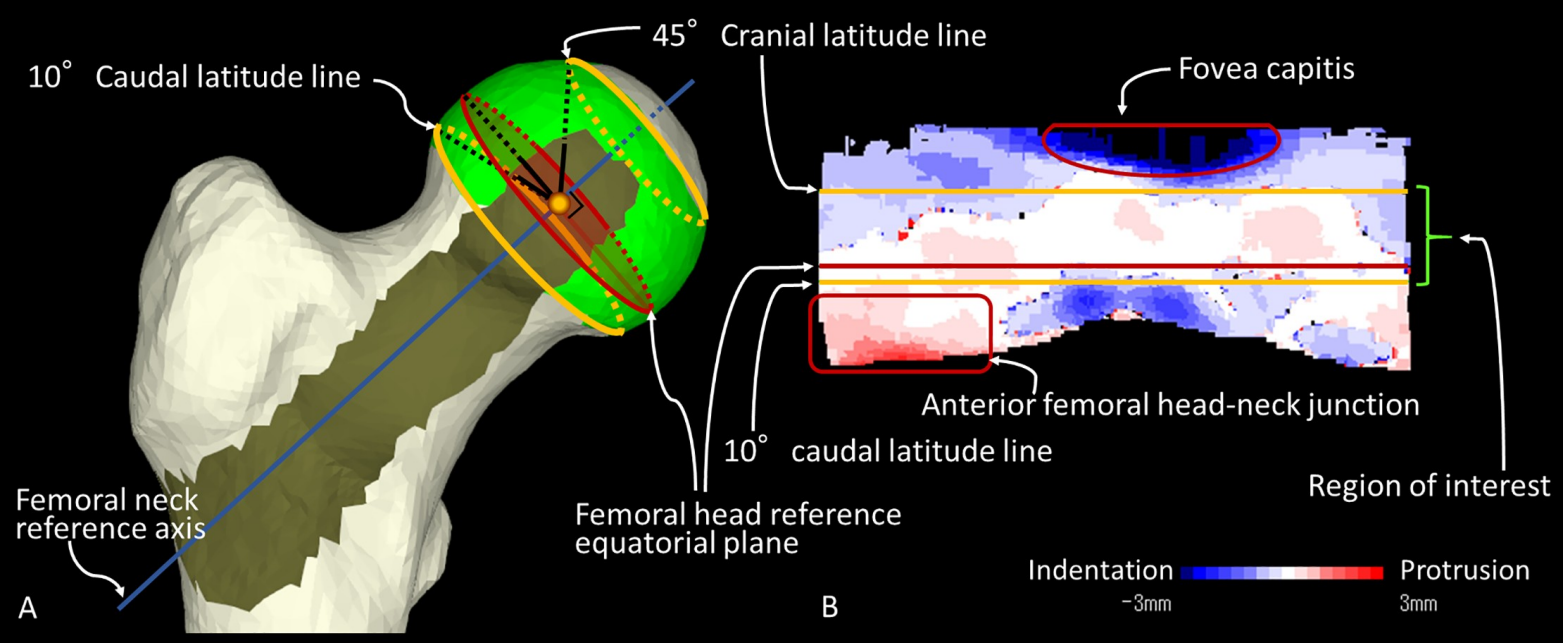
(A) Determination of the region of interest on the femoral head surface to calculate the 3D femoral head radius of curvature and femoral head center. (B) Atlas view of a planar projection of the femoral head surface, with the color map representing bony protrusion (red) or depression (blue).

The femoral head center and radius of curvature were calculated according to the technique described by Yanke et al. [24] Briefly, the calculation method was as follows: A point cloud model of the femoral head was created from the femoral point cloud model. The centroid of the femoral head was located and defined as an initial temporary femoral head center. The center of the cross section of the femoral neck was located and the line connecting this center and the initial temporary femoral head center was set as the femoral neck reference axis. The plane perpendicular to the femoral neck reference axis including the initial temporal femoral head center was set as the femoral head reference equatorial plane. In order to exclude the effects of the *fovea capitis* and the head-neck junction morphology on measurements, the region of interest (ROI) was set on the femoral surface spanning an arc from 45° cranial latitude to 10° caudal latitude based on the femoral head reference equatorial plane (Fig 1). The routine found the outmost point of the femoral head in the ROI, calculating its radial distances across the idealized sphere. Distances (and their standard deviation) between the temporary femoral head center and each point of the femoral head model were calculated. The temporary femoral head center hovered within a search range of ± 5.0 mm in 0.1 mm increments until the minimum standard deviation of the distance was achieved. This procedure was repeated until the smallest standard deviation of the distances was obtained. The temporary femoral head center with the smallest standard deviation of the distances was eventually defined as the definitive femoral head center. Femoral heads where the standard deviation of the distance between the definitive femoral head center and each point of the femoral head model exceeded 1.0 mm were labeled as aspherical femoral heads and excluded from the final evaluation. Based on the distances between this center and each point of the femoral head model, we calculated the three-dimensional (3D) femoral head radius of curvature using the same software.

A point cloud model of the lunate surface excluding the *fossa acetabuli* was created from the coxal bone point cloud model. The definitive femoral head center was defined as an initial temporary acetabular curvature center. Distances (and their standard deviations) between the temporary acetabular curvature center and each point of the lunate surface model were calculated. The temporary acetabular curvature center with the smallest distance standard deviation was defined as the definitive acetabular curvature center following the same procedure as the femoral head center. Based on distances between this curvature center and each point of the lunate surface model, the same algorithm as in the femoral head case was used to calculate the 3D acetabular radius of curvature.

#### 3D curvature mismatch ratio

The ratio of the 3D acetabular radius to the 3D femoral head radius was defined as the *curvature mismatch ratio*, an index of the curvature mismatch between acetabular curvature and femoral head curvature.

#### 3D center discrepancy

The distance between the acetabular curvature center and the femoral head center was defined as the *center discrepancy distance* aiming to evaluate joint eccentricity. Furthermore, we calculated the *center discrepancy direction* from the femoral head center to the acetabular curvature center by calculating its unit vector comprised of mediolateral, posteroanterior, and superoinferior direction components in reference to the body axis [25].

### Statistical analyses

The *chi* square test was used to compare categorical parameters among the three groups. The correlations between the 2D measurements and the 3D measurements of both the femoral head and acetabular radii were analyzed in each group respectively using Pearson’s correlation coefficient. The Pearson’s correlation (r) was graded as follows: ≥ +0.7 or ≤ −0.7 (very strong positive or very strong negative, respectively), +0.40 to +0.69 or −0.69 to −0.40 (strong positive or strong negative, respectively), +0.30 to +0.39 or −0.39 to −0.30 (moderate positive or moderate negative), +0.20 to +0.29 or −0.29 to −0.20 (weak positive or weak negative), +0.19 to −0.19 (no or negligible relationship) [26]. We compared statistically the 3D femoral head radius of curvature, the 3D acetabular radius of curvature, the 3D curvature mismatch ratio, and the 3D center discrepancy among the three groups using ANOVA with Tukey’s *post hoc* test. Statistical analyses were performed using JMP Pro 14 software (SAS Institute Japan, Tokyo, Japan). Data is presented as mean ± SD and the corresponding 95% confidence intervals. Significance was set at p < 0.05.

## Results

### Demographics

**Fig 2:**
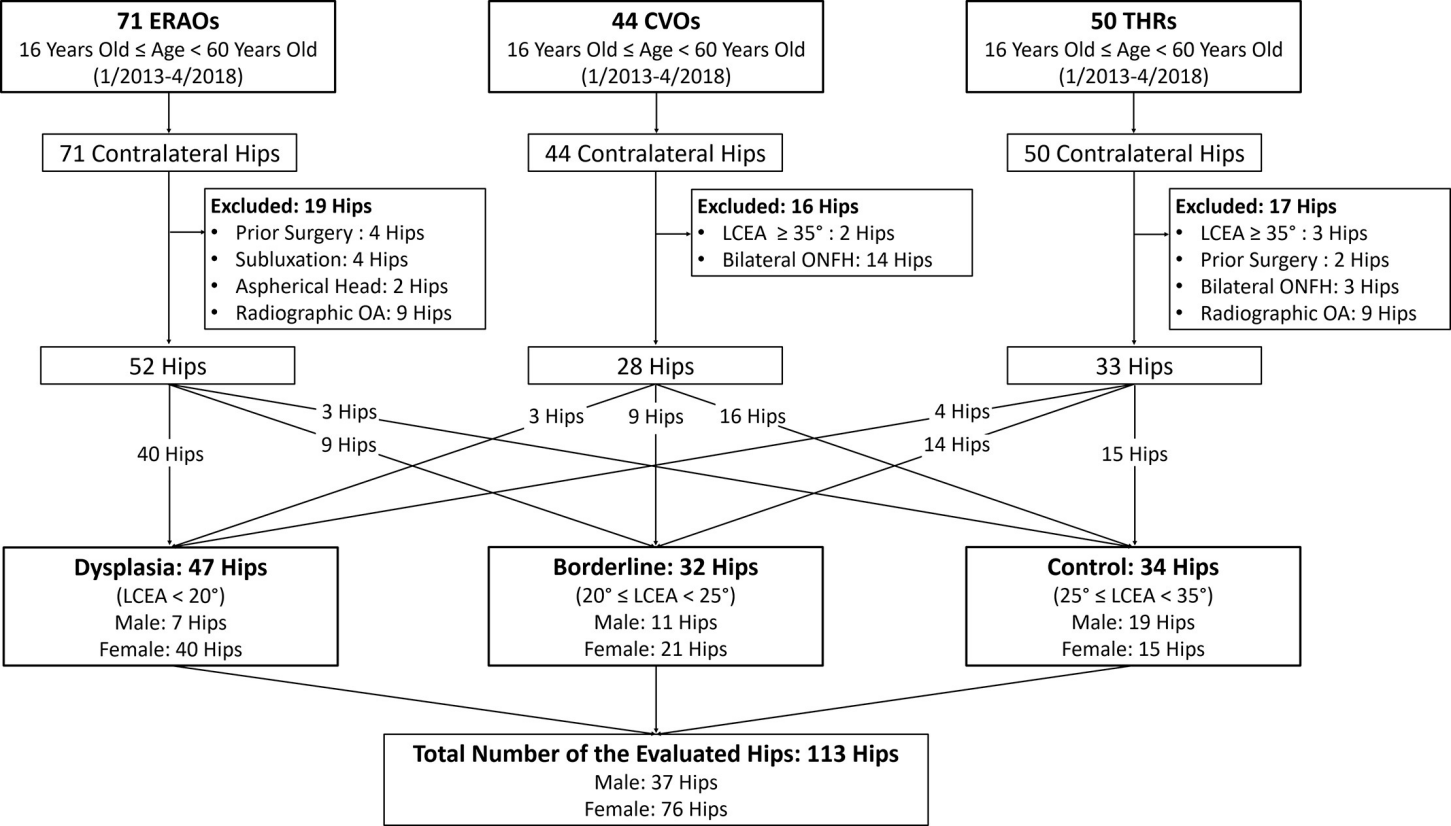
Flowchart of hip selection for evaluation, and the actual number of hips in each group. ERAO = eccentric rotational acetabular osteotomy, CVO = curved intertrochanteric varus osteotomy, THR = total hip replacement, OA = osteoarthritis, LCEA = lateral center-edge angle, ONFH = osteonecrosis of the femoral head.

ERAO, CVO, and THR were performed in 71 patients, 44 patients, and 50 patients respectively (Fig 2). Five hips had LCEA ≥ 35°, six hips had undergone prior hip surgery, four hips had subluxation, two hips had aspherical femoral heads, seventeen hips had ONFH, and eighteen hips had radiographic OA. Those fifty-two hips were excluded (Fig 2). The remaining one-hundred and thirteen hips were categorized as: dysplasia group, forty-seven hips; borderline group, thirty-two hips; and control group, thirty-four hips. In the dysplasia group, seven hips were from male patients, and forty hips were from female patients. In the borderline group, eleven hips were from male patients, and twenty-one hips were from female patients. In the control group, nineteen hips were from male patients, and fifteen hips were from female patients (Fig 2). No differences were noted among the three groups in weight or body mass index (BMI) with the available records. However, there were differences in the age, sex, or height (Table 1).

**Table 1:** Patient demographics in each group.

	Dysplasia (n = 47)	Borderline (n = 32)	Control (n = 34)	p value, overall
Age (years)	36 ± 13 (32–39)*	42 ± 14 (37–47)	44 ± 13 (40–49)	0.013
Sex (male /female)	7/40	11/21	19/15	0.001
Weight (kg)	57 ± 13 (53–61)	60 ± 14 (55–66)	62 ± 11 (58–66)	0.241
Height (cm)	159 ± 7 (157–161)*	162 ± 10 (158–165)	164 ± 7 (161–167)	0.015
BMI (kg/m^2^)	23 ± 5 (21–24)	23 ± 4 (22–24)	23 ± 3 (22–24)	0.920

Values of continuous parameters are expressed as mean ± SD with 95% confidence interval in parentheses

* significance compared with the control group; LCEA = lateral center-edge angle; BMI = body mass index.

### Radiologic parameters

The mean ARO of the borderline group (10.6 ± 4.3 [SD]°) was smaller than in the dysplasia group (19.3 ± 5.7°, p < 0.001) and larger than in the control group (3.4 ± 5.3°, p < 0.001). Although the mean acetabular anteversion angle of the borderline group (21.3 ± 3.7°) was larger than in the control group (18.0 ± 3.5°, p = 0.002), that of the borderline group was not different from the dysplasia group (23.3 ± 4.0°, p = 0.054) (Table 2).

**Table 2:** Radiologic parameters in each group.

	Dysplasia	Borderline	Control	p value		
				Borderline vs. Dysplasia	Borderline vs. Control	Dysplasia vs. Control
Lateral center-edge angle (°)	10.7 ± 5.1 (9.2–12.2)	23.3 ± 1.4 (22.8–23.9)	29.7 ± 5.5 (27.8–31.6)	< 0.001	< 0.001	< 0.001
Acetabular roof obliquity (°)	19.3 ± 5.7 (17.6–21.0)	10.6 ± 4.3 (9.0–12.2)	3.4 ± 5.3 (1.6–5.2)	< 0.001	< 0.001	< 0.001
Acetabular anteversion angle (°)	23.3 ± 4.0 (22.1–24.5)	21.3 ± 3.7 (19.9–22.6)	18.0 ± 3.5 (16.8–19.3)	0.054	0.002	< 0.001

Values are expressed as mean ± SD with 95% confidence interval in parentheses.

### Correlations between 2D and 3D measurements

For the femoral head radius, there were very strong positive correlations with 2D and 3D measurements in all groups (dysplasia; r = 0.852, p < 0.001, borderline; r = 0.887, p < 0.001, control; r = 0.914, p < 0.001). For the acetabular radius, although there was a very strong positive correlation with 2D and 3D measurements in the control group (r = 0.795, p < 0.001), there was a strong positive correlation in the borderline group (r = 0.564, p < 0.001) and there was a moderate positive correlation in the dysplasia group (r = 0.356, p = 0.014) (Table 3).

**Table 3:** 2D and 3D femoral head and acetabular radii of curvatures and correlations between 2D and 3D Measurements.

		Two-dimensional	Three-dimensional	Correlation coefficient
Femoral head radius of curvature	Dysplasia	22.0 ± 1.6 (21.5–22.4)	22.2 ± 1.4 (21.8–22.6)	r = 0.852, p < 0.001
	Borderline	22.5 ± 2.2 (21.8–23.3)	22.7 ± 1.8 (22.1–23.4)	r = 0.887, p < 0.001
	Control	22.7 ± 1.5 (22.2–23.2)	22.9 ± 1.5 (22.4–23.5)	r = 0.914, p < 0.001
Acetabular radius of curvature	Dysplasia	28.5 ± 3.7 (27.4–29.6)	27.3 ± 2.8 (26.5–28.1)	r = 0.356, p = 0.014
	Borderline	26.2 ± 2.7 (25.3–27.2)	25.6 ± 2.0 (24.8–26.3)	r = 0.564, p < 0.001
	Control	24.7 ± 2.1 (23.9–25.4)	24.5 ± 1.4 (23.9–25.0)	r = 0.795, p < 0.001

Values are expressed as mean ± SD with 95% confidence interval in parentheses.

### 3D Femoral head and acetabular radii of curvatures

**Fig 3:**
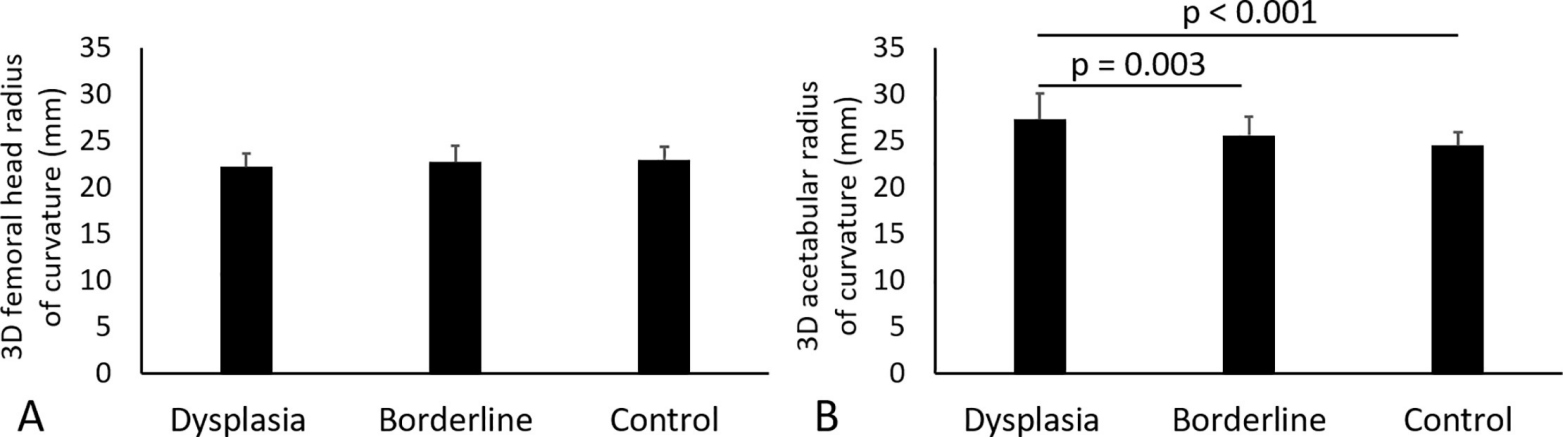
(A) 3D femoral head radius of curvature and (B) 3D acetabular radius of curvature in each group. Error bars span one SD.

Neither the dysplasia group (22.2 ± 1.4 [95% confidence interval {CI}, 21.8 to 22.6] mm, p = 0.281), nor the control group (22.9 ± 1.5 [95% CI, 22.4 to 23.5] mm, p = 0.821) mean 3D femoral head radii of curvature were different than the borderline group (22.7 ± 1.8 [95% CI, 22.1 to 23.4] mm). (Fig 3A). Although the mean 3D acetabular radius of curvature in the borderline group (25.6 ± 2.0 [95% CI, 24.8 to 26.3] mm) was not different from the control group (24.5 ± 1.4 [95% CI, 23.9 to 25.0] mm, p = 0.111), that of the borderline group was smaller than in the dysplasia group (27.3 ± 2.8 [95% CI, 26.5 to 28.1] mm, p = 0.003) (Fig 3B).

#### 3D Curvature mismatch ratio and 3d center discrepancy

**Fig 4:**
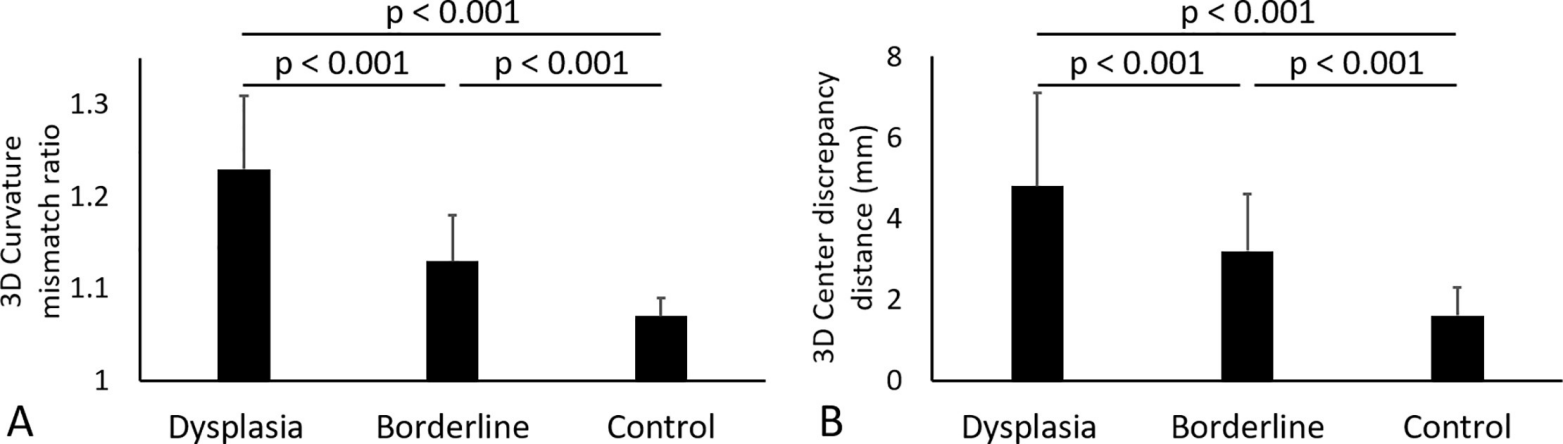
(A) 3D curvature mismatch ratio and (B) 3D center discrepancy distance in each group. Error bars span one SD.

**Fig 5:**
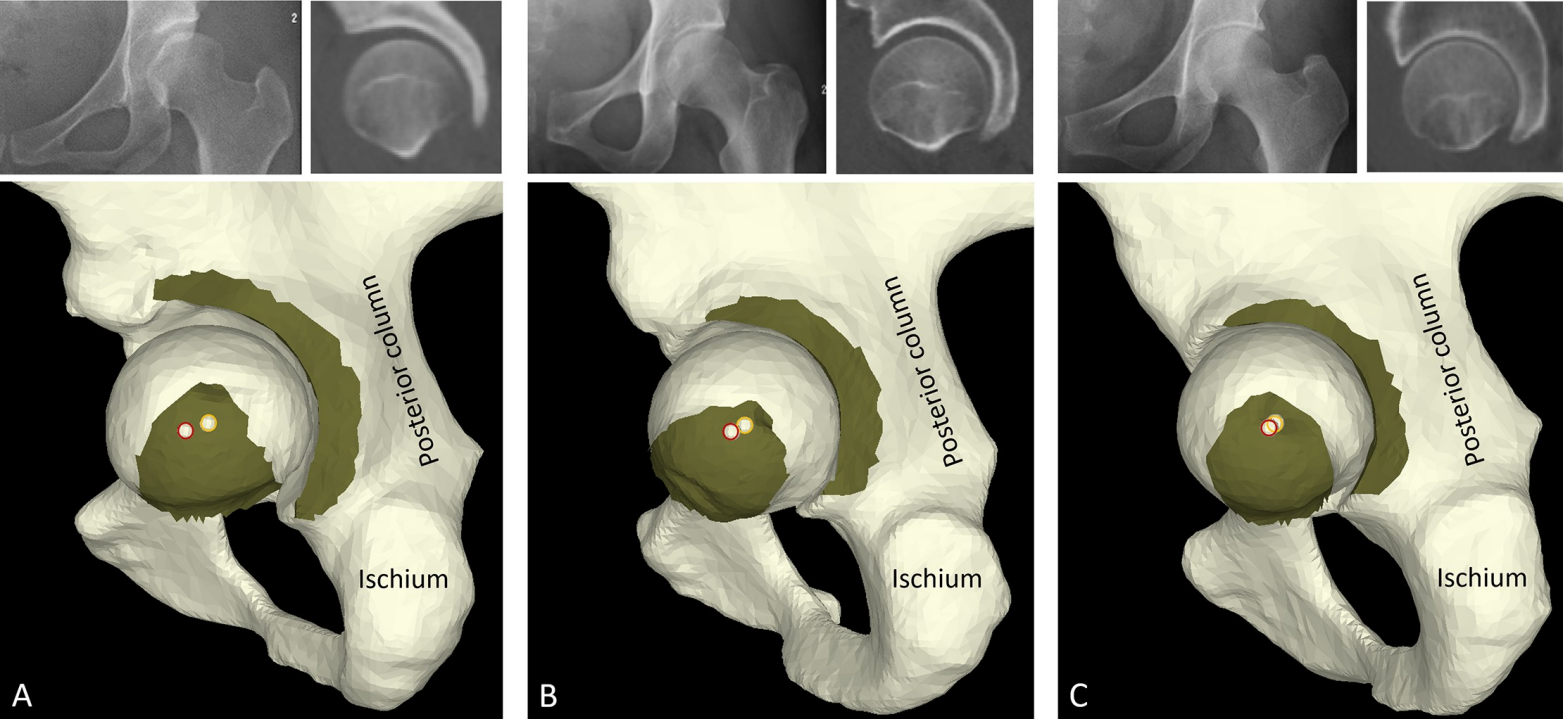
Representative AP pelvic radiographs, left hip mid-sagittal plane CT images, and reconstructed 3D models images with acetabular curvature center and femoral head center in each group. (A) Dysplastic hip, LCEA = 10.0°, 3D curvature mismatch ratio = 1.27, 3D center discrepancy distance = 6.13 mm. (B) Borderline dysplastic hip, LCEA = 22.3°, 3D curvature mismatch ratio = 1.14, 3D center discrepancy distance = 4.14 mm. (C) Control hip, LCEA = 32.4°, 3D curvature mismatch ratio = 1.07, 3D center discrepancy distance = 0.69 mm. The red circles denote the acetabular curvature center. The femoral head center is shown as a yellow circle. The posterior walls and femurs are partially resected in order to visualize the curvature mismatch between the acetabular curvature and the femoral head curvature, and the center discrepancy between the acetabular curvature center and the femoral head center.

The mean 3D curvature mismatch ratio in the borderline group (1.13 ± 0.05 [95% CI, 1.11 to 1.14]) was smaller than the dysplasia group (1.23 ± 0.08 [95% CI, 1.20 to 1.25], p < 0.001) and larger than the control group (1.07 ± 0.02 [95% CI, 1.06 to 1.07], p < 0.001) (Figs 4A and 5A–5C). The mean center discrepancy distance in the borderline group (3.2 ± 1.4 [95% CI, 2.7 to 3.7] mm) was smaller than the dysplasia group (4.8 ± 2.3 [95% CI, 4.1 to 5.5] mm, p < 0.001) and larger than the control group (1.6 ± 0.7 [95% CI, 1.3 to 1.8] mm, p < 0.001) (Figs 4B and 5A–5C).

Neither the dysplasia group (0.63 ± 0.18 [95% CI, 0.58 to 0.68], p = 0.703) nor the control group (0.59 ± 0.50 [95% CI, 0.41 to 0.76], p = 0.408) mean center discrepancy mediolateral direction showed differences with respect to the borderline group (0.69 ± 0.19 [95% CI, 0.62 to 0.76]). Although the mean center discrepancy posteroanterior direction in the borderline group (0.53 ± 0.24 [95% CI, 0.44 to 0.62]) was not different from the dysplasia group (0.61 ± 0.23 [95% CI, 0.54 to 0.68], p = 0.418), that of the borderline group was larger than in the control group (0.32 ± 0.38 [95% CI, 0.19 to 0.45], p = 0.012). Similarly, in both the dysplasia group (0.31 ± 0.24 [95% CI, 0.25 to 0.38], p = 0.474) and the control group (0.05 ± 0.43 [95% CI, -0.10 to 0.20], p = 0.098), the mean center discrepancy superoinferior direction was not different from the borderline group (0.23 ± 0.34 [95% CI, 0.10 to 0.35]; Table 4).

**Table 4:** Center discrepancy direction in each group.

Center discrepancy direction unit vector	Dysplasia	Borderline	Control	p value		
				Borderline vs. Dysplasia	Borderline vs. Control	Dysplasia vs. Control
Mediolateral (+: lateral, -: medial)	0.63 ± 0.18 (0.58–0.68)	0.69 ± 0.19 (0.62–0.76)	0.59 ± 0.50 (0.41–0.76)	0.703	0.408	0.827
Posteroanterior (+: anterior, -: posterior)	0.61 ± 0.23 (0.54–0.68)	0.53 ± 0.24 (0.44–0.62)	0.32 ± 0.38 (0.19–0.45)	0.418	0.012	< 0.001
Superoinferior (+: inferior, -: superior)	0.31 ± 0.24 (0.25–0.38)	0.23 ± 0.34 (0.10–0.35)	0.05 ± 0.43 (-0.10–0.20)	0.474	0.098	0.002

Values are expressed as mean ± SD with 95% confidence interval in parentheses.

## Discussion

The first finding in this study is that the correlations between the 2D and 3D acetabular radii measurements weaken depending on the severity of dysplasia although there are very strong positive correlations with the 2D and 3D femoral head radii measurements regardless of dysplasia severity. The second finding is that 3D curvature mismatch and 3D joint eccentricity between the acetabulum and the femoral head of the borderline dysplastic hip are smaller than those of frank dysplastic hip and larger than those of control hip.

Regarding the femoral head radius, there were very strong positive correlations with 2D measurements based on the AP pelvic radiograph and 3D measurements based on CT images in all groups. Since our primary outcome was to evaluate the curvature mismatch between the acetabulum and the femoral head, we measured the 2D femoral head radius using the method by Anderson et al. In this method, the spherical template is fitted to the aspect of the femoral head contained by the acetabulum because the head-neck junction deformity such as cam lesions can increase the radius [22]. Regarding the measuring of the 3D femoral head radius, we set the ROI based on our previous studies [23,24]. In order to exclude the effects of the fovea capitis and the head-neck junction morphology, where the possibility of a cam lesion is higher (as shown in the example of Fig 1), the ROI was set on the femoral surface spanning an arc from 45° cranial latitude to 10° caudal latitude based on the femoral head reference equatorial plane. The trustworthiness of the measurement methods that exclude the effects of the head-neck junction morphology, may be higher due to this fact. The measurement methods in both 2D and 3D, which exclude the head-neck junction from the ROI, may have enabled the very strong positive correlations in our study. As for the acetabular radius, the correlations between the 2D and 3D measurements were moderate positive in the dysplasia group, strong positive in the borderline group, and very strong positive in the control group. In other words, this result means that the more severe the dysplasia is, the more difficult it is to estimate the acetabular radius of curvature based on the AP pelvic radiograph alone. We believe that accurate acetabular radius evaluation requires a 3D method.

This study inquires if 3D curvature mismatch and joint eccentricity are different between borderline dysplastic hip and frank dysplastic or control hips. In order to evaluate the 3D joint congruity, two parameters were defined to answer that question: The 3D *curvature mismatch ratio* and the 3D *center discrepancy*. Joint congruity has been an important concept in considering OA risk factors [3,27,28]; however, there is no clear consensus on what “congruity” means. Additionally, commonly used hip congruity classifications have low intra-observer and inter-observer reliability [16,17]. Therefore, we believe that a more objective measure of 3D joint congruity is necessary.

**Fig 6:**
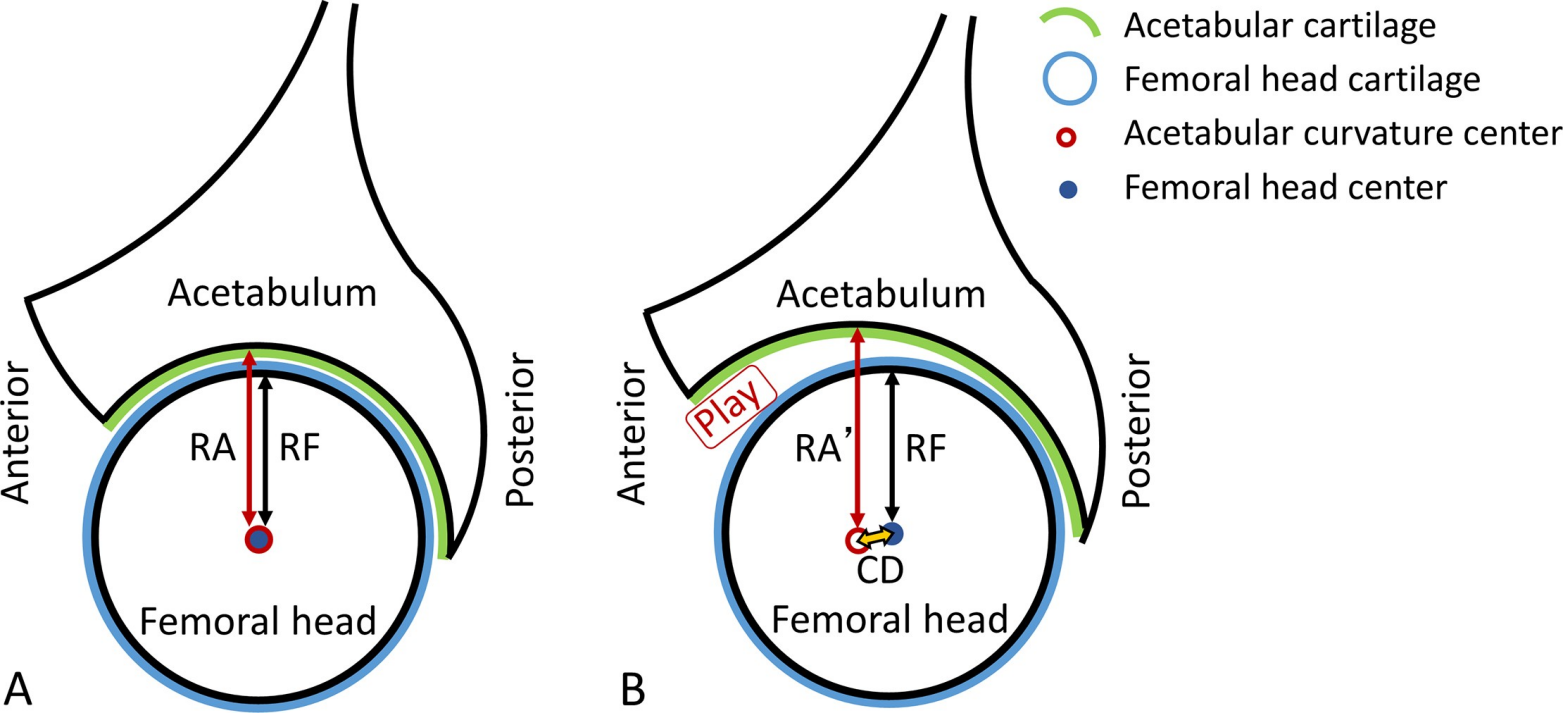
Diagrams of the theoretical relationship between curvature mismatch ratio and center discrepancy distance. (A) Congruent concentric congruent hip, (B) poorly congruent eccentric hip. RA, RA’: acetabular radius of curvature, RF: femoral radius of curvature, RA (RA’)/RF: curvature mismatch ratios, and CD: center discrepancy distance.

In a concentric congruent hip joint, the arc of the femoral head matches the arc of the acetabulum with a consistent joint space throughout [15–17]. In such a case, the ratio of the acetabular radius to the femoral head radius (curvature mismatch ratio) is close to unity. Additionally, the distance between the acetabular curvature center and the femoral head center (center discrepancy distance) is nearly zero (Fig 6A).

Only a small amount of clearance is required to reduce friction from a mechanical point of view (if there is appropriate lubrication) given the reduced contact points between surfaces. Generally, excessive clearance, which can be called “play”, can cause abnormal movement and this abnormal movement may result in instability. Regarding the hip joint, loss of congruity in the ball and socket joint idealized concept of the hip creates “play”. In theory, if both the curvature mismatch ratio and the center discrepancy distance were large, it is suggested that there is play between the femoral head surface and the acetabular surface in the joint and that the positional relationship between the femoral head center and the acetabular curvature center is eccentric (Fig 6B), leading to a less constrained unstable joint.

Our results showed that the 3D curvature mismatch ratio and the 3D center discrepancy distance of the borderline dysplastic hip are smaller than those of frank dysplastic hip and larger than those of control hip. While it is not clear what amount of clearance is appropriate and what amount of clearance is excessive for the hip joint, loss of joint congruity, as shown in the current study may be a structural indicator of joint instability in borderline dysplasia.

It should be noted that we used bone surfaces of the femoral head and acetabulum to evaluate joint congruity; therefore, the curvature mismatch ratio cannot reach the index value of unity since there are offset distances resulting from the presence of articular cartilage in both femoral head and acetabulum. In addition, this can be further affected by the fact that these articular cartilage layers do not have uniform thickness [29]. It would be ideal to use cartilage surfaces to evaluate curvature mismatch. However, a current practical limitation due to the use of clinical CT is its inability to visualize femoral and acetabulum cartilage surfaces. On the one hand, MRI can visualize the cartilage surface. Besides, recent developments in image analysis techniques have enabled 3D morphological analysis based on MRI both from other groups and our own research [30,31]. Further investigations, which are based on an evaluation method that considers cartilage thickness, such as MRI, are necessary to validate the present results. In addition, joint instability is a *dynamic phenomenon*, making future studies measuring joint motion a necessary condition to demonstrate an association between the new indices proposed in the present study and joint instability.

The center discrepancy directions of borderline group and dysplasia group were more anteriorly oriented than the control group. This direction difference seems to be caused by the larger mean acetabular anteversion angle in the borderline group or dysplasia group than the control group. Considering that LCEA increases in order of control group, borderline group, and dysplasia group, it is reasonable that the center discrepancy direction in the coronal plane becomes more lateral and superior, depending on the severity of dysplasia. Interestingly, our results indicate that the direction of the dysplasia group was more inferiorly oriented than the control group and that the mediolateral directions were not different among the three groups. In order to elucidate the difference in the center discrepancy direction, further studies are needed to evaluate local irregularity or distortion in the acetabulum and/or dynamic femoral head translation to the acetabulum.

We evaluated the 3D center discrepancy not as a direct sign of instability but as an *index of eccentricity* with hip neutral position. The evaluation was performed using CT images in the supine position with hips neutral position. Although the femoral head radius of curvature, the acetabular radius of curvature, and the 3D curvature mismatch ratio are theoretically robust to hip position when the CT images are acquired, the 3D center discrepancy distance or direction might be sensitive to the position within the scanner. The positional relationship between the femoral head and the acetabulum with hip neutral position was reported as not different between supine AP pelvic radiographs and weight-bearing conditions [32]. Additionally, this positional relationship is concentric within 30° of flexion in the normal hip [33]. Nevertheless, we have made effort to obtain the hip neutral position CT images. Due to the diversity of frank and borderline dysplastic hips, such as excessive femoral anteversion, it is difficult to identify the hip neutral position, especially neutral rotation, from only the hip joint images. Therefore, we carefully standardized the positioning of patients in the scanner. Furthermore, we also confirmed the hip neutral position based on both the entire femur and pelvis alignment, and the positional relationship between the pelvis and the knee joints using obtained CT images. However, the analysis using images obtained with upright and weight bearing is more ideal than that of supine position. Besides, in order to evaluate the hip instability based on femoral head translation, it is essential to evaluate the translations in multiple hip flexion angles and compare them, as the definition of joint instability is dynamic by nature. Further investigations are necessary for these issues.

Our results indicate that congruity of the borderline dysplastic hip is impaired but its incongruity is not as severe as the dysplastic hips. As for the borderline dysplasia, it is recognized that instability or concomitant cam deformity may cause labrum tears and cartilage degeneration [11–14,34,35]. Regarding the concomitant cam deformity, there are several reports that evaluated the 3D morphology in the borderline dysplastic hips [34–38]. Therefore, we focused on the objective measure of 3D joint congruity in this study. In our method, we evaluated the 3D femoral head radius of curvature, assuming that the femoral heads are spherical surfaces. Since cam deformities and aspherical femoral heads can theoretically affect the measured values, we did not include the head-neck junction in the ROI and excluded the aspherical femoral heads from the final evaluation. It should be noted that the aspherical femoral heads, mentioned here, are the heads we defined based on the standard deviation of the distance between the femoral head center and each point of the femoral head surface model. Therefore, the definition is different from that generally defined using the Mose template. Since joint congruity in hip with an aspherical femoral head is clinically very important [39], establishing a method for evaluating 3D joint congruity of the aspherical femoral heads is necessary for the future study. Besides, it is difficult to characterize whether the pathological mechanism of the symptomatic borderline dysplastic hip is instability, concomitant impingement, or both [40]. Certainly, more investigation is warranted on this issue.

A few limitations need to be considered with this study. First, there is a potential risk of selection bias. Since it would be unethical to perform CT scans on healthy subjects for research, we used the contralateral hips CT data of subjects who underwent imaging for preoperative examinations of ERAO, CVO, or THR. We unified the cohort into the consecutive patients who underwent surgery in the same period at our institution. On the other hand, we did not include trauma patients in the cohort, because we could not get their standardized AP pelvic radiographs and CT images in the supine position with hip neutral position. As hip dysplasia is epidemiologically more common among females, there was a difference in the intragroup ratio of male versus female and the mean height in the dysplasia group was shorter than that in the control group. The differences in mean body height among groups can potentially affect the femoral head and the acetabular radii. Therefore, we propose the 3D curvature mismatch ratio, which was the primary outcome from this study, as a way to normalize in order to exclude the effects of the individual physical constitution. By definition, the curvature mismatch ratio and the center discrepancy direction are not affected by the individual’s physical constitution. However, the center discrepancy distance can be sensitive to body habitus. As for the center discrepancy distance, further investigations on the effect of the individual physical constitution, such as sex and height, on the measured values are necessary for clinical use. Second, symptoms, physical examinations, and clinical scores were not considered in this study. Since our primary outcome was to evaluate the bony morphology, we focused on the objective measure of the individual inherent bony morphology. Further studies are needed to evaluate an association between the current results and symptoms or physical examinations. Third, the radiologic parameters were measured by a single reader and the reliability has not been evaluated in this study. It has been reported that the inter- and intra-observer reliability were excellent for ARO and fair to good for LCEA [41,42]. It is recognized that since we should determine the center of the femoral head to measure LCEA, the reliability of LCEA is slightly worse than that of ARO [43]. Since the method of Anderson *et al*. was reported to have higher reliability than the conventional method, we performed measurements using this method in identifying the center of the femoral head [22].

In conclusion, we showed that the correlation between the 2D and 3D acetabular radii measurements weakens depending on the severity of dysplasia and that both the 3D curvature mismatch ratio between the acetabulum and femoral head, and the 3D center discrepancy distance between the acetabular curvature center and the femoral head center in the borderline group, were larger than in the control group and smaller than in the dysplasia group. The present results demonstrated that three-dimensional congruity of the borderline dysplastic hip is impaired but its incongruity is not as severe as the dysplastic hips. The indices of the 3D curvature mismatch ratio and the 3D center discrepancy that we proposed in this study can be valuable signs of joint congruity in patients with borderline dysplasia. However, future studies are necessary to clarify an association between the new indices and OA pathogenesis in borderline dysplasia.

## Supporting information

S1 Data (XLSX)
